# Predicting Older Adults’ Mobile Payment Adoption: An Extended TAM Model

**DOI:** 10.3390/ijerph20021391

**Published:** 2023-01-12

**Authors:** Cheng-Chia Yang, Shang-Yu Yang, Yu-Chia Chang

**Affiliations:** 1 Department of Healthcare Administration, Asia University, Taichung 41354, Taiwan; 2 Department of Long Term Care, National Quemoy University, Kinmen County 892009, Taiwan

**Keywords:** mobile payment, older adults, behavioral intention, influence factor

## Abstract

This study adopted an advanced model, combining the technology acceptance model, the theory of reasoned action, the diffusion of innovations, trust, and five aspects of perceived risk, to measure the factors that influence the behavioral intentions of older adults to use mobile payments. A total of 365 questionnaires were collected from older adults aged 55 years or older from 20 community care sites in central Taiwan. Partial least-squares structural equation modeling was used to test our research model. The results showed that attitude was the main determinant of M-payment in older adults. Moreover, increasing the usefulness, ease of use, and observability of M-payment helped older adults improve their attitudes toward M-payment, thereby increasing their intention to use it. Trust had a significant effect on the usefulness and ease of use of M-payment, while the main factors affecting trust were only performance and financial risks.

## 1. Introduction

Mobile payment (M-payment), a service that uses mobile devices and wireless communication technologies for business transactions [1], possesses many advantages over traditional payment methods. In addition to the speed and convenience of its purchase mechanism, M-payment services allow users to encrypt transaction data, thereby increasing their reliability [2]. One would expect that M-payment services would increase merchants’ transaction volumes, reduce transaction costs, and increase customer loyalty [3]. However, although M-payment is a remarkable technology, the growth in consumer usage is rather slow [4], with several consumers still using cash as their payment method [5].

Research shows that compared with other age groups, the likelihood of older adult groups using the internet is still low [6]. In particular, it has been pointed out that the rate at which young people under the age of 25 use M-payment is 11 times that of those over the age of 65 [7]. Yet, even if older adults’ mobile phone usage increases, it will not necessarily mean that they are willing to use M-payment, as most older adult individuals prefer to use cash for transactions [8]. In addition, older adults’ low acceptance of these applications [9] may have resulted from the quality and effectiveness of the applications, the accuracy of information provided, the fear of operational errors, and the privacy and security of personal data [10,11].

A review of the existing literature reveals that there is scarcely any literature that addresses the willingness of older adults to use M-payment [8,12,13]. To the best of our knowledge, most of the previous articles on M-payment have considered consumers to be a uniform sample. Francisco et al. mentioned that age has a significant moderating effect on the intention to use M-payment [14]. However, their study used 35 years old as the age cut-off point, which fails to provide insight into the intentions of older age groups, hence the need for a study focusing on older age groups.

In order to analyze older adults’ behavior regarding the adoption of innovative technology, several behavioral decision theories and intentional models have been developed and reported in the scientific literature. The technology acceptance model (TAM) has been regarded as the most robust, parsimonious, and influential model of innovation acceptance behavior [15,16], and therefore, we considered this theoretical model as a basis for the purpose of the present study. This study aimed to explore in detail older adults’ intentions to use M-payment with the expectation of providing businesses and governments with factors to consider when promoting the use of M-payment among older adults.

## 2. Theoretical Background and Hypotheses

Dahlberg et al. mentioned that M-payment had previously been studied based on factors such as perceived usefulness (PU), perceived ease of use (PEOU), trust, risk, and security [17]. For instance, Lian and Li found that consumer motivation to use M-payment is influenced by factors of PU, convenience, online promotions or preferential offers, and social identity [18]. However, most consumer barriers to using M-payment include a lack of trust and security, the complexity of the system, and the habit of paying in cash [18,19]. Further, when people switch from cash to M-payment, perceived security and privacy are significant factors considered [20]. Cham et al. mentioned several factors that affect the use of M-payment services among older adults, including functional aspects (e.g., perceived complexity, perceived incompatibility, and perceived cost), psychological aspects (e.g., lack of trust and technology anxiety), and risks (i.e., privacy risks, security risks, financial risks, and functional risks) [8].

Based on the above discussion, it could be concluded that trust and risk in M-payment are common focuses of current research on M-payment for the older adult population. In addition, this study suggested that social influence is a factor that should not be overlooked when assessing technology acceptance by older adults [10,21]. Therefore, in this study, the theory of reasoned action (TRA’s) subjective norms and the diffusion of innovation theory (DOI’s) observability and social image were added to discuss the determinants of older adults’ intentions to use M-payment.

### 2.1. Influence of Attitudes on Behavioral Intentions

The main dependent variable in TAM research is the intention to use, defined as the likelihood that an individual will use a particular technology [22]. According to the TAM, the main key influence on the intention to use is a person’s attitude toward using a technology [15], which refers to the degree to which an individual places a positive or negative emphasis on the technology. Attitudes and behavioral intentions have been validated in more recent studies [23,24], which should be applied to older adults’ intentions to use M-payment. This study hypothesized that older adults’ attitudes toward M-payment services determine their intention to use M-payment. In particular, with M-payment being an uncommon new technology for most older adults, it may be better to explain the use intention of this group of users through their attitudes toward M-payment than through ease of use and usability. Therefore, the following hypothesis was proposed:

**H1:**  *Older adults’ attitudes toward M-payment positively affect intention to use*.

### 2.2. Effects of PU and PEOU on Attitudes

According to Davis et al., the two most important factors in exploring the TAM are PU and PEOU [15]. On the one hand, we have PU, the extent to which an individual believes that the use of technology will improve their quality of life. PEOU, on the other hand, is the extent to which an individual believes that the use of technology requires effort. PU directly and indirectly predicts behavioral intentions through attitudes toward the use of technology [10,25,26,27]. Many studies have also confirmed that although M-payment applications offer many benefits, older adults may find them difficult to learn and use, which in turn affects their attitudes and behavioral intentions [10,25,26,27]. Thus, it was suggested that PEOU directly or indirectly predicts behavioral intentions to use technology through attitudes toward its use [28]. In addition, the TAM also considers PU and PEOU, which is treated as a predictor of PU, a relationship supported by many studies [29,30]. Therefore, the following hypotheses were proposed:

**H2:**  *PU positively affects older adults’ attitudes toward the use of M-payment*.

**H3:**  *PEOU positively affects older adults’ attitudes toward the use of M-payment*.

**H4:**  *PEOU positively affects older adults’ PU of the use of M-payment*.

### 2.3. Subjective Norms in Relation to Behavioral Intentions

Subjective norms refer to the self-perceptions of an individual regarding whether people important to them (e.g., family and friends) support their use of a new system [22]. The TRA identifies subjective norms and attitudes as determinants of behavioral intentions [15]. Many studies have empirically demonstrated that subjective norms significantly affect older adults’ intentions to use technology products [10,21,31]. Therefore, this study suggests that older adults may be motivated by peers or friends to use M-payment and proposes the following hypothesis:

**H5:**  *Subjective norms positively affect older adults’ intentions to use M-payment*.

### 2.4. Effect of Image on Attitudes toward M-Payment

The concept of “image” is derived from the theory of DOI. Moore and Benbasat defined “image” as the extent to which people perceive that the use of innovation enhances their image or status in the social system [32]. In particular, they found that it influences people’s willingness to use innovative technologies [32]. Past research has found that image has an important influence on attitudes [33]. This finding means that individuals may perceive that the use of innovative technology leads to an enhanced personal image, improving their attitudes toward the use of the technology [34,35]. Noteworthily, negative images can also produce negative attitudes. For instance, Huang and Chang studied the intentions of older adults to use a walker and found that most of the older adults in their study felt that their personal image was damaged as a result of using the technology, resulting in a significant negative effect on their attitudes toward using it [36]. Therefore, this study argues that enhanced image after older adults receive attention and admiration from their peers or others for using M-payment during transactions reinforces their attitudes toward M-payment.

**H6:**  *Image positively affects older adults’ attitudes toward using M-payment*.

### 2.5. Effect of Observability Attitudes toward M-Payment

Observability, also derived from the theory of DOI [37], refers to the extent to which an innovation can be observed before it is used [32]. Karahanna et al. combined observability with the TAM in their study and confirmed that it impacts attitudes toward IT use [38]. Similarly, it has been found in studies discussing the intention to use mobile banking that higher observability has a positive effect on individuals’ attitudes toward using mobile banking [39]. Therefore, it was hypothesized that when older adults regularly see positive results of M-payment usage in the population, they will perceive its benefits and develop a more positive attitude toward M-payment.

**H7:**  *Observability positively affects older adults’ attitudes toward M-payment*.

### 2.6. Relationship of Trust with PU and PEOU

Trust is an important critical success factor for online transactions such as e-commerce and internet banking [40]. Consumers are often reluctant to engage in online trading activities due to a lack of trust in the transaction process [41]. Similarly, trust is a significant factor for M-payment [42]. Past research has defined trust as an individual’s belief that a supplier will perform certain activities according to the latter’s expectations [43]—an expectation that others will be honest in providing promised services. This presumption indicates that one party expects that the other party’s words or promises are reliable and that the other party will fulfill his or her obligations in the exchange relationship (Francisco, Juan, and Francisco, 2014).

Furthermore, trust is significantly and positively related to PU [42,44,45]. Yang pointed out that consumer expectations of quality and functional consistency influence consumers’ trust in mobile shopping [30]. This finding suggests that suppliers need to provide consumers with more accurate and timely information about mobile shopping and provide more reliable and personalized mobile shopping services to satisfy consumers. These expectations may improve the user experience and enhance consumers’ perceptions of the system. Therefore, it is suggested that trust is related to PU [46,47,48] in that older adults do not perceive M-payment to be a useful method for everyday transactional activities unless they have confidence in its operating mechanisms.

There is also a significant positive correlation between trust and PEOU [47,49]. When people’s trust in the M-payment system is higher, they spend less time understanding and learning how M-payment works, allowing them to apply M-payment to transactions without much effort [14]. Therefore, the current study points out that trust is related to PEOU since it reduces the effort required to perform M-payment. Hence, this study proposed the following hypotheses:

**H8:**  *Trust positively affects older adults’ PU of M-payment*.

**H9:**  *Trust positively affects older adults’ PEOU of M-payment.*

### 2.7. Relationship between Perceived Risk and Trust

Perceived risk is considered a negative factor affecting consumers’ trust in M-payment [50]. It refers to a consumer’s expectation of loss associated with making an M-payment transaction, which includes personal information leakage as well as the loss of funds [51]. Past studies have identified that perceived risk could be considered a necessary condition for trust [14,43,50]. In terms of the causal relationship between trust and perceived risk, this study considers trust to have a greater influence on older adults’ behavioral intentions to use M-payment. Therefore, perceived risk is an antecedent of trust. A higher perceived risk would reduce older adults’ trust in the functionality and benefits of mobile payments. This study classified perceived risks into privacy, time, financial, performance, and psychological risks. The following discussion addresses the relationship between these types of risks and trust.

#### 2.7.1. Relationship between Perceived Privacy Risk and Trust

Dinev and Hart argue that privacy represents the control of interpersonal transactions, with the ultimate goal of enhancing autonomy or minimizing risks [52]. Meanwhile, the privacy risk of M-payment refers to the concern that individuals may have that they could lose their privacy due to the disclosure of personal information to external transaction vendors. Such perceived privacy risks can affect the trustworthiness of mobile transactions. Further, many studies have confirmed the relationship between trust and perceived privacy risk [53,54]. Therefore, it was assumed that older adults’ trust in the technology would be reduced due to the higher perceived privacy risk involved in M-payment. Hence, this study proposed the following hypothesis:

**H10:**  *Perceived privacy risk negatively affects older adults’ trust in M-payment*.

#### 2.7.2. Relationship between Perceived Performance Risk and Trust

Perceived performance risk refers to consumers’ perceived loss if a product or service fails to meet or exceed their expectations [55]. It is particularly concerned with whether the product functions as expected. Chang et al. distinguished three groups of M-payment users based on their behavioral intentions and found that perceived performance risk is an important risk factor common to all groups of users [56]. This study suggests that older adults making transactions through M-payment may not be able to touch or feel the reality of the transaction before and after the transaction. This speculation means that older adults may not know whether M-payment will work as expected or not, which further increases the perceived performance risk [57]. Therefore, this study suggested that perceived performance risk has a significant effect on trust [56,58]. Hence, the following hypothesis was proposed:

**H11:**  *Perceived performance risk negatively affects older adults’ trust in M-payment*.

#### 2.7.3. Relationship between Perceived Financial Risk and Trust

Perceived financial risk represents the possibility of monetary loss resulting from the use of M-payment [59]. In particular, financial risk is a common element of risk in the said technology, in which the uncertainty involved in authorizing M-payment may increase users’ concerns [60]. Bashir et al. suggested that many financial risk issues affect consumers’ trust in online shopping providers, such as lost credit cards [61], no refund guarantees, hidden charges, loss of money, and sales fraud. Thus, people are concerned that their credit card information may be stolen when making transactions or that they may lose money through repeated debits. Therefore, this study suggests that perceived financial risk significantly impacts older adults’ trust in M-payment, as they may be worried about losing money through network transactions [62]. Hence, this study proposed the following hypothesis:

**H12:**  *Perceived financial risk negatively affects older adults’ trust in M-payment*.

#### 2.7.4. Relationship between Perceived Psychological Risk and Trust

Perceived psychological risk refers to the likelihood that an individual will experience emotional stress as a result of their behavior [59]. It can be defined as the possible loss of self-esteem or ego due to the frustration of not achieving one’s desired goals [51]. This type of psychological risk is often associated with inexperience, as consumers unfamiliar with the system’s operation are more susceptible to psychological stress resulting from the fear of making poor choices [58,63]. Furthermore, Hong and Cha mention that perceived psychological risk and trust are closely related [63]. Their study suggests that in order to increase consumers’ trust in online suppliers, it is necessary to meet the latter’s psychological needs in their online shopping experiences, such as providing easy processes for the return of goods for an exchange or refund, which would reduce their psychological stress.

Therefore, this study suggests that older adults are so accustomed to traditional cash transactions that they may experience frustration and anxiety when transactions fail using M-payment. This psychological risk may also affect the trust of older adults in M-payment. Therefore, this study proposed the following hypothesis:

**H13:**  *Perceived psychological risk negatively affects older adults’ trust in M-payment*.

#### 2.7.5. Relationship between Perceived Time Risk and Trust

Perceived time risk includes consumers’ fear of additional time pressures resulting from switching from an existing transaction method to a new one [63]. Therefore, it can be inferred that when older adults consider switching from the cash payment method to an M-payment method, they may expect they will experience longer transaction times due to their unfamiliarity with the system or need additional time to familiarize themselves with the M-payment system. They may also fear that they are unable to learn, as learning becomes more difficult as the brain ages. These situations are time risk factors that affect consumers [57]. Therefore, this study suggests that time risk affects older adults’ desire to learn and increases their reluctance to change from traditional transactions, decreasing their trust in M-payment. Hence, this study proposed the following hypothesis:

**H14:**  *Perceived time risk negatively affects older adults’ trust in M-payment*.

## 3. Materials and Methods

### 3.1. Sample and Procedure

This study is a cross-sectional study in which a structured questionnaire was adopted to quantify and analyze the samples. Older adults aged 55 years or older were taken as the research subjects. The survey was conducted from October to November 2020. There were 408 community care sites in central Taiwan, at each of which an average of 15–30 older adults gathered. This study randomly selected 20 community care sites to validate the research model. Four research assistants assisted in distributing the questionnaires, visiting the sites to explain the purpose of the study, and conducting the sample collection. To encourage participation in this study, those who wished to take the survey were given a voucher worth TWD 100. With the participant’s consent, an introductory video (e.g., https://youtu.be/XN6JxmfhKHw, accessed on 2 October 2020) of M-payment was played—an approach widely adopted by international researchers in recent years [64]. These participants were then briefed on the terms and definitions used in the questionnaire. Participants were also encouraged to ask the research assistant questions in case of confusion before answering the questionnaire. This approach would help eliminate the possibility of participants not understanding the content of the survey. Of the total 430 questionnaires sent out for this study, 65 invalid questionnaires were excluded. This brought the total to 365 questionnaires included in the analysis, with a recovery rate of 86.9 percent, which met the sample size requirement. The study was reviewed and approved by the institutional review board (IRB) of the Taichung Jen-Ai Hospital (IRB Protocol No.: 10927; approval date: 14 May 2020).

### 3.2. Questionnaire Scale and Variable Measurement

The scale to investigate older adults’ intentions to use M-payment in this study included 9 constructs developed based on relevant research. First, the scales of *PU*, *PEOU*, Attitudes, and Behavioral Intention were developed based on the questionnaire developed by Wang and Chou [65]. There were 12 questions with 3 questions for each construct, such as “Do you think using M-payment could help you find better deals?”, “Do you think it would be easy to learn how to use M-payment?”, “Do you have a positive view toward using M-payment?”, and “Do you plan to continue to use M-payment in the future?” Second, the evaluation scales for Observability and Image were developed by referring to Moore and Benbasat [32]. There were four questions in total for Observability, such as “Do you see many people around you using M-payment?”, and there were three questions in total for Image, such as “In a group, people who use M-payment are more highly respected.” Further, the scale for Subjective Norms was made based on Kim et al., with a total of three questions [66], including “Do people important to you think you should use M-payment?” On the other hand, the scale for *Trust* was based on Gefen et al. [45], with a total of four questions, including “Based on your past experience of using M-payment, do you know it is trustworthy?” and “Based on your past experience using M-payment, do you know it is reliable?” Finally, Perceived Risk was based on the five risk factors (privacy, performance, psychology, finance, and time) described by Pavlou [16]. There were 3 questions for each factor for a total of 15 questions. All items were scored according to a 5-point Likert scale, with strongly disagree, disagree, neutral opinion, agree, and strongly agree rated on a scale of 1 to 5.

### 3.3. Data Analysis

This study used SEM to perform statistical analyses. SEM has received much criticism for establishing causal relations from associations [67]. However, using SEM to test model structures still has credibility [67]. The hypotheses of model structure causal relations in this study were derived from scientific knowledge and logical arguments confirmed by previous research findings. As such, this study’s hypotheses were based on a well-founded, research-based theoretical model. SEM can still be used as a statistical method to test this study’s hypotheses.

This study mainly used partial least-squares structural equation modeling (PLS-SEM) to construct predictive models, allowing a causal model analysis between potential variables, which is suitable for models with both formative and reflective variables and superior to general linear structural relationship models. It is particularly suitable for exploratory studies considering that not only does it accept single-item dimensions, but it also has good predictive and explanatory power regardless of variable distribution patterns and the sample size [68]. PLS-SEM can detect both paths (structural model) and factors (measurement model) in one model. In addition, PLS-SEM combines factor analysis with the minimum hypothesis of proximity regression analysis. The resulting R-squared value represents the extent to which the independent variables can explain the dependent variables. SmartPLS was the software used to analyze the measurement model and structural model. In this study, the Bootstrap Resampling Method (BRM) was used to calculate and infer the parameters from a sample of 5000 [69]. Since the BRM is a nonparametric statistical inference method, good results can also be obtained for a small-sized sample.

## 4. Results

### 4.1. Demographics and Characteristics

In the basic data of the sample in this study, the majority, comprising 186 participants, accounting for 51 percent, were males, and 179 were females (49%). In terms of age, 180 participants (49.3%) were aged 55–60 years, followed by 110 (30.1%) aged 61–65 years, 71 (19.5%) aged 66–70 years, and 4 (1.1%) aged 80 years or older. The overall mean age of the subjects was 61.8 years with a standard deviation of 7.9, with the lowest age being 55 years and the highest being 81 years. Most participants (142, 38.9%) had an educational level of elementary school or below, followed by 104 (28.5%) participants with a junior high school educational level and 74 (20.3%) participants with a senior high school educational level. About 39 percent of the survey participants had used M-payment before, with 169 participants (84.4%) using EasyCard or iPASS Card, followed by 11 (4.7%) using LINE Pay, 16 (6.8%) using Apple Pay, and 9 (3.8%) using Google Pay.

The survey data in this study came from a single source (i.e., each questionnaire was answered by only one participant), which may lead to Common Method Variance (CMV) bias. Therefore, to confirm the presence of CMV bias, this study used Harman’s one-way test [70] to extract five factors with eigenvalues greater than one in the unrotated condition. The cumulative explained variance was 75.4 percent, while the explained variance in the first factor was 41.5 percent and less than 50 percent. In particular, it was tentatively determined that the effect of CMV was not significant.

### 4.2. Reliability and Validity of Research Instruments

In order to prevent the problem of high collinearity, this study partly observed the value of the Variance Inflation Factor (VIF), where VIF > 10 means high collinearity [71]. The VIF values of each item in this study are shown in Table 1. In order to confirm the fitness of the model, the standardized root-mean-square residual (SRMR) value was used to assess the research model’s fitness. The model has good fitness when the SRMR of the Saturated Model and the Estimated Model is less than 0.08, while a score of less than 0.1 for both models is in the acceptable range [72]. Meanwhile, the SRMR of the Saturated Model and the Estimated Model = 0.074, indicating that the research model has good fitness.

In this study, three indicators, Individual Item Reliability, the Composite Reliability (CR) of potential variables, and the Average Variance Extracted (AVE) of potential variables, were used to assess the measurement patterns of reflective indicators, as suggested by Bagozzi and Yi [73]. The indicators are explained as follows. Individual Item Reliability assesses the factor loading of the measurement variable on the potential variable and tests the statistical significance of each variable loading. All factor loading values in this study were above the recommended value of 0.5, indicating significance. The coefficients of factor loading in the sample ranged from 0.594 to 0.974, as shown in Table 1.

The CR of the potential variables is the composition of the reliability of all of the measurement variables, indicating the constructs’ internal consistency. The higher the reliability, the higher the internal consistency of these potential variables. Chin suggests a value of 0.7 or higher [74]. As shown in Table 1, the CR values of the variables in the model exceeded the standard of 0.7, while the CR values in this study ranged from 0.906 to 0.963, indicating the good internal consistency of the research model. The AVE of the potential variables is calculated as the explanatory power of the variance in the potential variables. Fornell and Larcker suggested that the standard value of AVE should be greater than 0.5 [75]. Table 1 shows that the AVE values of all variables in the study model were higher than the standard value of 0.5. Therefore, the reflective variables in this study have good convergent validity.

Finally, in this study, the method for measuring the discriminant validity was used to observe the square root of the AVE. If the square root of the AVE is greater than the correlation coefficient of other homogeneous constructs, the degree of the relationship between potential constructs is less than the degree of the relationship within the construct, indicating the model’s discriminant validity. The basis for this study is that the square root of the AVE of the potential variables must be greater than the correlation matrix of the other different constructs. The results indicate that the values of the square root of the AVE were all greater than the correlation coefficient of each construct (Table 2), which means that the constructs have discriminant validity. From the above analysis, the reliability, convergent validity, and discriminant validity of the constructs in this study are at acceptable levels.

### 4.3. Mediation Regression Models of Study Variables

**Figure 1:**
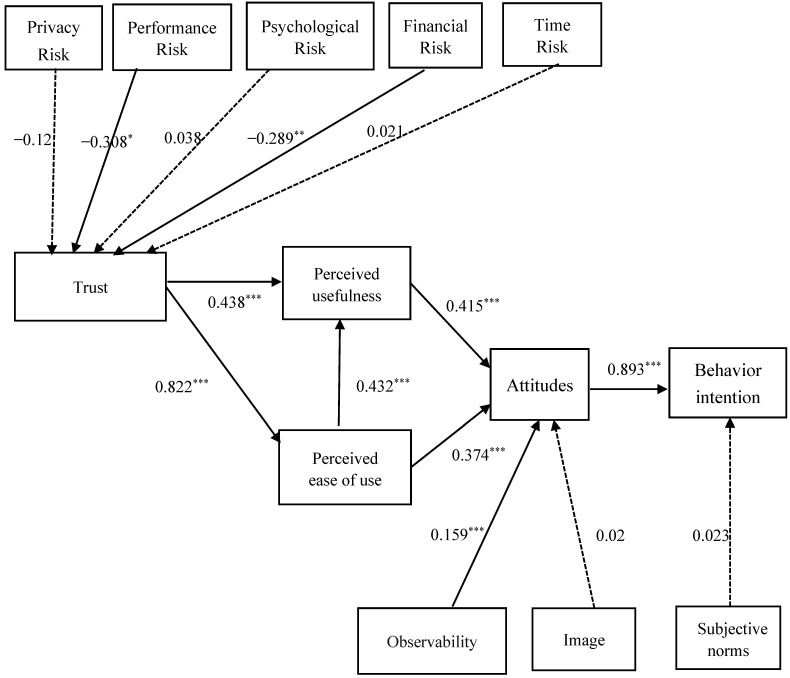
Estimated model architecture. * *p* < 0.05; ** *p* < 0.01; *** *p* < 0.001.

This study employed the Bootstrap Resampling Method in PLS to check and estimate the significance of the paths in the structural model, in which R^2^ is the main index for judging the model’s goodness of fit [74]. The path relationships among the constructs were estimated using PLS, and the standardized values were adopted as the path values. All hypotheses testing the path relationships of the studied models reached the significance level of α = 0.05. The path analysis coefficients of the structural model are shown in Table 3 and Figure 1.

Older adults’ attitudes toward M-payment had a positive effect on their intention to use it (β = 0.893, t = 39.82, *p* < 0.001), so H1 was established. This study found that the more positive the attitude, the higher the intention to use. A total of 82.4 percent of the variation in intention to use was explained. PU had a positive effect on attitudes (β = 0.415, t = 7.42, *p* < 0.001), so H2 was established. PEOU had a positive effect on attitudes (β = 0.374, t = 7.058, *p* < 0.001). Therefore, H3 was established, indicating that when the older adults perceived M-payment as useful and easy to use, it affected their attitudes toward M-payment. In addition, PEOU had a positive effect on PU (β = 0.432, t = 6.478, *p* < 0.001), thereby establishing H4. Among the constructs related to social influence, observability had a positive effect on attitudes (β = 0.159, t = 4.139, *p* < 0.001); hence, H7 was established, indicating that the higher visibility of M-payment had a positive effect on attitudes toward M-payment, which explained a total of 73.5 percent of the variance in attitudes. However, image had no significant effect on attitudes (β = 0.02, t = 0.543, *p* > 0.05); hence, H6 was not established. Finally, subjective norms also had no effect on behavioral intention to use M-payment (β = 0.023, t = 0.765, *p* > 0.05); hence, H5 was not established.

In addition, trust had a positive effect on PU and PEOU (β = 0.438, t = 6.85, *p* < 0.001; β = 0.822, t = 38.84, *p* < 0.001). Thus, H8 and H9 were established. This finding indicates that the higher the trust of older adults in M-payment, the higher the PU and PEOU of M-payment. Trust explained 66.6 percent of the variance in PEOU and 69.5 percent of the variance in PU. Furthermore, this study analyzed the relationship between the five constructs of perceived risk and trust and found that only performance risk and financial risk had a significant effect on trust (β = −0.308, t = 2.351, *p* < 0.05; β = −0.289, t = 3.04, *p* < 0.01). Thus, both H11 and H12 were established. This finding indicates that older adults’ perception of M-payment was that, one, products and services purchased through M-payment would disappoint, and two, that there is a risk of financial loss due to an M-payment transaction going wrong, both of which had a significant effect on trust, explaining a total of 7.7 percent of the variance in trust. However, privacy risk, psychological risk, and time risk did not have a significant effect on trust (β = −0.21, t = 1.942, *p* >0.05; β = 0.038, t = 0.431, *p* >0.05; β = 0.021, t = 0.464, *p* >0.05). Moreover, the model’s predictive power was also examined by computing the cross-validated redundancy index (Q2) for the endogenous variable. All Q2 values for the model varied significantly above zero (BI = 0.722; AT = 0.635; PU = 0.544; PEOU = 0.536; Trust = 0.068), indicating the high predictive capacity of the exogenous constructs [74].

## 5. Discussion

Many past articles on technology acceptance have used the TAM as their theoretical framework [76]. However, because the TAM excludes demographic factors and external variables, this study developed and validated a conceptual model that incorporates the DOI’s observability and image, the TRA’s subjective norms, and trust and perceived risk to discuss which factors are important for older adults’ behavioral intentions to use M-payment. This study showed that 9 out of 14 hypotheses were confirmed.

### 5.1. Theoretical Implications

This study confirmed that trust could influence PU and PEOU, consistent with past studies [42,45,47]. This trust had to be gained by surmounting the perceived performance risk and perceived financial risk. This outcome can be expected because, in an M-payment environment, the main concern of older adults in using this type of service is whether their service needs will be met and whether unnecessary financial risks will be incurred. These premises mean that M-payment service providers must ensure that users experience the desired benefit of using the functions of the M-payment service while reducing the possibility of financial losses (e.g., repeated debits), as the fear of monetary loss is an important factor to overcome in gaining users’ trust and willingness to use M-payment [61]. When M-payment service providers sufficiently prevail over the above risk perceptions of older adults, users will begin to trust the providers and the technology, thus promoting their perception of the usefulness and ease of use of M-payment and increasing their intention to use it [44,49].

Furthermore, no significant relationship between privacy risk and trust was found, which contradicts the findings of previous articles [53,54]. However, there is a growing concern about the extent to which individuals can protect their personal information. Therefore, privacy risks continue to raise concerns in e-commerce articles. Moreover, older adults are less information-literate [77] and are even less knowledgeable about information security and privacy hazards [78]. Under such circumstances, information literacy and information security knowledge affect the perceptions of privacy risks [79]. Thus, they are more concerned about whether the M-payment service will run as expected and not incur extra costs due to inadvertent operation. Therefore, privacy risks are not as important as performance and financial risks.

Psychological risk had no impact on trust, possibly due to the maturity of M-payment technology in Taiwan and the fierce competition among a large number of M-payment platform vendors, which has improved the quality of many M-payment systems and services [80]. Furthermore, these M-payment system platforms are not only stable but also user-friendly with easy-to-use interfaces that have significantly reduced users’ psychological anxiety about M-payment operation errors. Therefore, the psychological risk is less of a barrier to trust in M-payment. In terms of time risk, it was originally hypothesized that older adults might be reluctant to change their traditional payment methods because they would feel that their time was too valuable to waste on this learning curve [81], thereby leading to their distrust in M-payment. However, it was found that time risk did not have a significant effect on trust. Therefore, it is tentatively concluded that the popularity of M-payment platforms in Taiwan, coupled with the maturity of the functionality, allows for faster transaction processing [80]. In addition, the convenience of M-payment and the resulting efficiency enhance users’ ability to make mobile purchases, helping them save a lot of time [51]. Therefore, perceived time risk did not affect trust.

This study found a positive effect of observability on attitudes, similar to that of previous studies [82,83], suggesting that having the opportunity to observe a technology in use provides users with more confidence before actually using it for themselves. For instance, when older adults see more people use M-payment technology and it has higher observability, they will develop positive attitudes toward using the technology. However, image did not appear to have a significant effect on attitude, a finding that differs from that of several previous studies [33,36]. Thus, this study suggests that image may not influence attitude because older adults tend to ignore the effects of social pressure, image, and social status and are only inclined to pursue more emotionally meaningful goals [84]. Finally, no significant effect of subjective norms was found on behavioral intentions, probably because the statistical results of this study suggest that attitude explains about 82.4 percent of the variance in behavioral intentions. In this case, individuals’ behavioral intentions are commonly influenced by their attitudes [85,86], which may moderate the influence of subjective norms on behavioral intentions, a finding similar to those of past studies [87].

### 5.2. Practical Implications

This study sought to understand the determinants influencing the intention to use M-payment among older adults over the age of 55. The results indicate that for older adults, attitude was an important factor that influenced the intention to use M-payment. The user’s feelings or attitude determined the propensity to use the new technology. The strength of the influence of attitude may be due to the novelty of the technology service, as users may not have enough information to make an informed judgment on its use. Therefore, the first step in increasing older adults’ intentions to use the technology is to change their attitudes toward M-payment. PEOU and PU can help improve the attitudes of older adults toward M-payment. These are the two key factors determining the success of older adults’ adoption of M-payment.

There are many barriers to the use of these innovative technologies for older adults, including concerns about the quality and effectiveness of the applications, the accuracy of the information provided, fear of operational errors, and concerns about privacy and the security of personal data [10,11]. Nevertheless, the benefits of M-payment can be emphasized through educational approaches. It is also important to teach older adults how to use the technology to enhance their PEOU and PU. This strategy may help reduce the anxiety of older adults toward the technology [88], improve their attitudes toward M-payment, and indirectly increase their intention to use it. In addition, M-payment service providers should focus on developing the functions of payment tools to make them more useful, speedier, and convenient. It is suggested that service providers convey the unique benefits of M-payment, such as security, trustworthiness, and on-demand transactions at any time and any place, through more promotions and advertisements. They should also try to find ways to get older adults to move beyond traditional methods (cash and credit cards) and recognize new methods that have been implemented in the market. By stimulating more consumers to use M-payment, its observability would be increased and result in an increase in PU. Furthermore, the easier it is for older adults to see the outcomes of new technologies, the more likely they are to adopt them [37].

Trust is an essential factor in enhancing PU and ease of use, and overcoming perceived performance risk and perceived financial risk is an important antecedent to influencing older adults’ trust in mobility payments [57,62]. This finding suggests that in order for M-payment services to grow in the older adult market segment, M-payment providers should make older users aware of the benefits of M-payment usage. Older adults are particularly concerned about the possibility of losing money using M-payment. For instance, personal credit card information may be stolen when making transactions, or unnecessary fees may be incurred due to a failed operation. Therefore, providers should assure users by developing strategies to reduce perceived risk and increase confidence in the system, especially by offering refund guarantees to reduce the risk involved in falling victim to fraudulent transactions. Older adults’ trust in M-payment is likely to increase when they perceive that the M-payment provider is acting in their best interest. This mechanism promotes the PU and PEOU of M-payment in older adults and increases their intention to use it [44].

### 5.3. Research Limitations and Future Research Directions

Despite the theoretical and practical implications of this study, several limitations may require more in-depth investigation. First, the convenience sampling method used in this study may reduce the representativeness of the results. Therefore, it is recommended that the findings be treated with caution. Future studies could compare rural and urban areas, as the geographic location may lead to different results in terms of the information literacy of the participants recruited. Additionally, demographic distinctions, such as gender and educational level, should be made for older adults, as these factors may lead to differences in information literacy [89]. Second, although this study used cross-sectional data collection with a survey through a structured questionnaire, it may be necessary to observe behavioral outcomes from a long-term perspective to understand the trends and changes in user behavior. Finally, because this study was voluntary, individuals could freely choose to participate. This limitation introduced the risk of self-selection bias, whereby recruiting more individuals with previous exposure to M-payment services means they may have more information about the technology that affected their responses. Nevertheless, most participants in this study sample were not users of the M-payment service, and thus, this may not have significantly affected the study.

## 6. Conclusions

A theoretical model was developed in this study to discuss the behavioral intentions influencing the use of M-payment by older adults (those over the age of 55). As few past studies had addressed the behavioral intentions of older adults to use M-payment, this study incorporated five dimensions of perceived risk to more fully assess the model of M-payment use intentions. The results suggest that attitude is an important factor in an older adult’s decision to adopt M-payment. The perceived usefulness and ease of use of M-payment were the predecessors that influenced attitudes. Trust also had a significant effect on the usefulness and ease of use of M-payment. Furthermore, while perceived risks did affect trust, performance and financial risks were the only main factors affecting trust.

This study suggests that for older adults to have a higher level of trust in M-payment, it is essential to ensure that service functions have the intended benefits and that the financial losses incurred are reduced. However, this study also found that previous studies on e-business have often mentioned that there was no significant relationship between privacy risks and trust. This might be due to insufficient information literacy among older adults (e.g., lack of knowledge about information security and privacy hazards). This finding is worthy of in-depth exploration in future research. Moreover, increasing the observability of M-payment helps improve the attitudes of older adults toward M-payment, thereby increasing their intention to use it. Finally, subjective norms did not positively affect behavioral intentions, possibly due to the attitude variable. It is possible that the effect of interpersonal relationships may be moderated because older adults place more importance on their own attitudinal preferences for M-payment rather than merely following a reference group.

This study provides businesses and governments with factors to consider when promoting the use of M-payment among older adults. It also allows practitioners to better understand the perceived risks of M-payment, which can be used to design the risk mitigation strategies and trust-building mechanisms necessary for developing new applications or finding ways to cater to this demographic segment of the market.

## Figures and Tables

**Table 1:** Reliability and convergent validity of measures.

Construct	Variable	Loading	T-Value	CR	AVE	Cronbach’s Alpha	VIF
Perceived Usefulness	PU1	0.896	65.512	0.923	0.799	0.875	2.792
	PU2	0.880	68.519				1.957
	PU3	0.904	80.160				2.840
Perceived Ease of Use	PEOU1	0.897	69.060	0.931	0.818	0.889	2.671
	PEOU2	0.917	87.952				2.801
	PEOU3	0.898	71.457				2.364
Attitudes	AT1	0.935	115.321	0.953	0.870	0.926	3.738
	AT2	0.931	98.261				3.442
	AT3	0.932	108.898				3.527
Behavioral Intention	BI1	0.943	93.095	0.957	0.882	0.933	4.175
	BI2	0.939	122.137				3.871
	BI3	0.936	84.284				3.678
Observability	VB1	0.867	68.210	0.906	0.708	0.862	2.289
	VB2	0.854	35.810				2.254
	VB3	0.793	24.389				2.234
	VB4	0.853	33.899				1.754
Image	IM1	0.911	72.748	0.933	0.824	0.893	2.972
	IM2	0.908	65.349				3.607
	IM3	0.903	50.201				3.620
Subjective Norms	SN1	0.928	69.257	0.943	0.847	0.910	2.966
	SN2	0.917	70.840				3.377
	SN3	0.917	85.572				2.881
Trust	TR1	0.905	75.920	0.942	0.802	0.918	3.737
	TR2	0.881	60.503				2.990
	TR3	0.899	70.085				3.257
	TR4	0.900	72.026				4.067
Privacy Risk	PVR1	0.876	4.838	0.948	0.859	0.927	4.066
	PVR2	0.865	4.484				4.785
	PVR3	0.868	4.548				4.742
Performance Risk	PR1	0.733	4.483	0.900	0.751	0.864	2.464
	PR2	0.841	7.195				2.234
	PR3	0.900	6.676				2.871
Financial Risk	FR1	0.905	22.726	0.937	0.833	0.900	2.535
	FR2	0.881	20.976				2.838
	FR3	0.938	34.08				3.689
Psychological Risk	PHR1	0.891	9.097	0.937	0.831	0.903	4.488
	PHR2	0.884	8.085				4.718
	PHR3	0.899	9.969				2.072
Time Risk	TR1	0.801	3.862	0.886	0.724	0.891	2.465
	TR2	0.805	3.373				2.966
	TR3	0.826	4.484				2.578

**Table 2:** Correlation matrix of potential constructs of measurement model.

	1	2	3	4	5	6	7	8	9	10	11	12	13
1	**0.894**												
2	0.792	**0.904**											
3	0.801	0.795	**0.933**										
4	0.775	0.778	0.798	**0.939**									
5	0.562	0.521	0.595	0.565	**0.841**								
6	0.513	0.487	0.476	0.464	0.384	**0.908**							
7	0.549	0.536	0.644	0.598	0.643	0.489	**0.920**						
8	0.784	0.818	0.805	0.810	0.583	0.439	0.587	**0.905**					
9	−0.053	−0.115	0.034	−0.044	−0.014	−0.065	−0.084	−0.053	**0.915**				
10	−0.209	−0.209	−0.198	−0.190	−0.148	−0.066	−0.210	−0.186	0.753	**0.841**			
11	−0.088	−0.168	−0.155	−0.141	−0.151	−0.022	−0.199	−0.193	0.729	0.744	**0.913**		
12	−0.192	−0.187	−0.170	−0.155	−0.166	−0.066	−0.257	−0.151	0.701	0.799	0.731	**0.912**	
13	−0.074	−0.136	−0.069	−0.071	−0.035	−0.021	−0.131	−0.072	0.758	0.757	0.767	0.724	**0.886**

Note: 1. PU; 2. PEOU; 3. Attitudes; 4. Behavioral Intention; 5. Observability; 6. Image; 7. Subjective Norms; 8. Trust; 9. Privacy Risk; 10. Performance Risk; 11. Financial Risk; 12. Psychological Risk; 13. Time Risk. Note 2: The square roots of the AVE values are shown in bold.

**Table 3:** Results of research hypotheses.

Hypothesis	Path Relationship	Path Coefficient	T Value	Result
H1	AT → BI	0.893	39.820 ***	Established
H2	PU → AT	0.415	7.420 ***	Established
H3	PEOU → AT	0.374	7.058 ***	Established
H4	PEOU → PU	0.432	6.478 ***	Established
H5	SN → BI	0.023	0.765	Not established
H6	Image → AT	0.020	0.543	Not established
H7	Observability → AT	0.159	4.139 ***	Established
H8	Trust → PU	0.438	6.854 ***	Established
H9	Trust → PEOU	0.822	38.848 ***	Established
H10	Privacy Risk → Trust	−0.120	1.942	Not established
H11	Performance Risk → Trust	−0.308	2.351 *	Established
H12	Financial Risk → Trust	−0.289	3.040 **	Established
H13	Psychological Risk → Trust	0.038	0.431	Not established
H14	Time Risk → Trust	0.021	0.464	Not established

Note: PU = perceived usefulness; PEOU = perceived ease of use; BI = behavioral intention; AT = attitudes; SN = subjective norms. Significance levels: * *p* < 0.05; ** *p* < 0.01; *** *p* < 0.001.

## Data Availability

The data that support the findings of this study are available on request from the corresponding author. The data are not publicly available due to privacy or ethical restrictions.

## References

[1] Dahlberg T., Mallat N., Ondrus J., Zmijewska A. (2008). Past, present and future of mobile payments research: A literature review. Electron. Commer. Res. Appl..

[2] Schwiderski-Grosche S., Knospe H. (2002). Secure mobile commerce. Electron. Commun. Eng. J..

[3] Slade E.L., Williams M.D., Dwivedi Y.K. (2013). Mobile payment adoption: Classification and review of the extant literature. Mark. Rev..

[4] Johnson V.L., Kiser A., Washington R., Torres R. (2018). Limitations to the rapid adoption of M-payment services: Understanding the impact of privacy risk on M-Payment services. Comput. Hum. Behav..

[5] Verkijika S.F. (2020). An affective response model for understanding the acceptance of mobile payment systems. Electron. Commer. Res. Appl..

[6] Magsamen-Conrad K., Upadhyaya S., Joa C.Y., Dowd J. (2015). Bridging the divide: Using UTAUT to predict multigenerational tablet adoption practices. Comput. Hum. Behav..

[7] Li B., Hanna S.D., Kim K.T. (2020). Who uses mobile payments: Fintech potential in users and non-users. J. Financ. Couns. Plan..

[8] Cham T.H., Cheah J.H., Cheng B.L., Lim X.J. (2021). I Am too old for this! Barriers contributing to the non-adoption of mobile payment. Int. J. Bank Mark..

[9] Rasche P., Wille M., Bröhl C., Theis S., Schäfer K., Knobe M., Mertens A. (2018). Prevalence of health app use among older adults in Germany: National survey. JMIR Mhealth Uhealth.

[10] Hoque R., Sorwar G. (2017). Understanding factors influencing the adoption of mHealth by the elderly: An extension of the UTAUT model. Int. J. Med. Inform..

[11] Kruse C.S., Mileski M., Moreno J. (2017). Mobile health solutions for the aging population: A systematic narrative analysis. J. Telemed. Telecare.

[12] Choudrie J., Junior C.-O., McKenna B., Richter S. (2018). Understanding and conceptualising the adoption, use and diffusion of mobile banking in older adults: A research agenda and conceptual framework. J. Bus. Res..

[13] Hanif Y., Lallie H.S. (2021). Security factors on the intention to use mobile banking applications in the UK older generation (55+). A mixed-method study using modified UTAUT and MTAM-with perceived cyber security, risk, and trust. Technol. Soc..

[14] Francisco L.-C., Juan S.-F., Francisco M.-L. (2014). Antecedents of the adoption of the new mobile payment systems: The moderating effect of age. Comput. Hum. Behav..

[15] Davis F.D., Bagozzi R.P., Warshaw P.R. (1989). User acceptance of computer technology: A comparison of two theoretical models. Manag. Sci..

[16] Pavlou P.A. (2003). Consumer acceptance of electronic commerce: Integrating trust and risk with the technology acceptance model. Int. J. Electron. Commer..

[17] Dahlberg T., Guo J., Ondrus J. (2015). A critical review of mobile payment research. Electron. Commer. Res. Appl..

[18] Lian J.W., Li J. (2021). The dimensions of trust: An investigation of mobile payment services in Taiwan. Technol. Soc..

[19] Nguyen H.V. (2018). Cash or cashless? Promoting consumers’ adoption of mobile payments in an emerging economy. Strateg. Dir..

[20] Loh X.M., Lee V.H., Tan G.W.H., Ooi K.B., Dwivedi Y.K. (2020). Switching from cash to mobile payment: What’s the hold-up?. Internet Res..

[21] Soh P.Y., Heng H.B., Selvachandran G., Anh L.Q., Chau H.T.M., Son L.H., Abdel-Baset M., Manogaran G., Varatharajan R. (2020). Perception, acceptance and willingness of older adults in Malaysia towards online shopping: A study using the UTAUT and IRT models. J. Ambient. Intell. Humaniz. Comput..

[22] Venkatesh V., Morris M.G., Davis G.B., Davis F.D. (2003). User acceptance of information technology: Toward a unified view. MIS Q..

[23] Bruner G.C., Kumar A. (2005). Explaining consumer acceptance of handheld Internet devices. J. Bus. Res..

[24] Schierz P.G., Schilke O., Wirtz B.W. (2010). Understanding consumer acceptance of mobile payment services: An empirical analysis. Electron. Commer. Res. Appl..

[25] Guner H., Acarturk C. (2020). The use and acceptance of ICT by senior citizens: A comparison of technology acceptance model (TAM) for elderly and young adults. Univers. Access Inf. Soc..

[26] Askari M., Klaver N.S., van Gestel T.J., van de Klundert J. (2020). Intention to use medical apps among older adults in the Netherlands: Cross-sectional study. J. Med. Internet Res..

[27] Mitzner T.L., Boron J.B., Fausset C.B., Adams A.E., Charness N., Czaja S.J., Dijkstra K., Fisk A.D., Rogers W.A., Sharit J. (2010). Older adults talk technology: Technology usage and attitudes. Comput. Hum. Behav..

[28] Revythi A., Tselios N. (2019). Extension of technology acceptance model by using system usability scale to assess behavioral intention to use e-learning. Educ. Inf. Technol..

[29] Tsai T.H., Lin W.Y., Chang Y.S., Chang P.C., Lee M.Y. (2020). Technology anxiety and resistance to change behavioral study of a wearable cardiac warming system using an extended TAM for older adults. PLoS ONE.

[30] Yang S. (2016). Role of transfer-based and performance-based cues on initial trust in mobile shopping services: A cross-environment perspective. Inf. Syst. E-Bus. Manag..

[31] Schepers J., Wetzels M. (2007). A meta-analysis of the technology acceptance model: Investigating subjective norm and moderation effects. Inf. Manag..

[32] Moore G.C., Benbasat I. (1991). Development of an instrument to measure the perceptions of adopting an information technology innovation. Inf. Syst. Res..

[33] Lin H.C., Chang T.Y., Kuo S.H. (2018). Effects of social influence and system characteristics on traceable agriculture product reuse intention of elderly people: Integrating trust and attitude using the Technology Acceptance Model. J. Res. Educ. Sci..

[34] Lin C.P., Bhattacherjee A. (2010). Extending technology usage models to interactive hedonic technologies: A theoretical model and empirical test. Inf. Syst. J..

[35] Venkatesh V., Davis F.D. (2000). A theoretical extension of the technology acceptance model: Four longitudinal field studies. Manag. Sci..

[36] Huang S.W., Chang T.Y. (2020). Social image impacting attitudes of middle-aged and elderly people toward the usage of walking aids: An empirical investigation in Taiwan. Healthcare.

[37] Rogers E.M., Singhal A., Quinlan M.M. (2014). Diffusion of Innovations. An Integrated Approach to Communication Theory and Research.

[38] Karahanna E., Straub D.W., Chervany N.L. (1999). Information technology adoption across time: A cross-sectional comparison of pre-adoption and post-adoption beliefs. MIS Q..

[39] Püschel J., Mazzon J.A., Hernandez J.M.C. (2010). Mobile banking: Proposition of an integrated adoption intention framework. Int. J. Bank Mark..

[40] Beldad A., De Jong M., Steehouder M. (2010). How shall I trust the faceless and the intangible? A literature review on the antecedents of online trust. Comput. Hum. Behav..

[41] Bryce J., Fraser J. (2014). The role of disclosure of personal information in the evaluation of risk and trust in young peoples’ online interactions. Comput. Hum. Behav..

[42] Dutot V. (2015). Factors influencing near field communication (NFC) adoption: An extended TAM approach. J. High Technol. Manag. Res..

[43] Khalilzadeh J., Ozturk A.B., Bilgihan A. (2017). Security-related factors in extended UTAUT model for NFC based mobile payment in the restaurant industry. Comput. Hum. Behav..

[44] Dutot V., Bhatiasevi V., Bellallahom N. (2019). Applying the technology acceptance model in a three-countries study of smartwatch adoption. J. High Technol. Manag. Res..

[45] Gefen D., Karahanna E., Straub D.W. (2003). Trust and TAM in online shopping: An integrated model. MIS Q..

[46] Choi J.K., Ji Y.G. (2015). Investigating the importance of trust on adopting an autonomous vehicle. Int. J. Hum.-Comput. Interact..

[47] Schnall R., Higgins T., Brown W., Carballo-Dieguez A., Bakken S. (2015). Trust, perceived risk, perceived ease of use and perceived usefulness as factors related to mHealth technology use. Stud. Health Technol. Inform..

[48] Talwar S., Dhir A., Khalil A., Mohan G., Islam A.N. (2020). Point of adoption and beyond. Initial trust and mobile-payment continuation intention. J. Retail. Consum. Serv..

[49] Hansen J.M., Saridakis G., Benson V. (2018). Risk, trust, and the interaction of perceived ease of use and behavioral control in predicting consumers’ use of social media for transactions. Comput. Hum. Behav..

[50] Yang Y., Liu Y., Li H., Yu B. (2015). Understanding perceived risks in mobile payment acceptance. Ind. Manag. Data Syst..

[51] Marriott H.R., Williams M.D. (2018). Exploring consumers perceived risk and trust for mobile shopping: A theoretical framework and empirical study. J. Retail. Consum. Serv..

[52] Dinev T., Hart P. (2004). Internet privacy concerns and their antecedents-measurement validity and a regression model. Behav. Inf. Technol..

[53] Mutimukwe C., Kolkowska E., Grönlund Å. (2020). Information privacy in e-service: Effect of organizational privacy assurances on individual privacy concerns, perceptions, trust and self-disclosure behavior. Gov. Inf. Q..

[54] Ozturk A.B., Nusair K., Okumus F., Singh D. (2017). Understanding mobile hotel booking loyalty: An integration of privacy calculus theory and trust-risk framework. Inf. Syst. Front..

[55] Tandon U., Kiran R., Sah A.N. (2018). The influence of website functionality, drivers and perceived risk on customer satisfaction in online shopping: An emerging economy case. Inf. Syst. e-Bus. Manag..

[56] Chang W.-L., Chen L.-M., Hashimoto T. (2021). Cashless Japan: Unlocking influential risk on mobile payment service. Inf. Syst. Front..

[57] Choi Y., Choi H. (2017). Risk factors affecting trust and satisfaction in mobile payment systems. Int. Inf. Inst. (Tokyo) Inf..

[58] Hong I.B., Cha H.S. (2013). The mediating role of consumer trust in an online merchant in predicting purchase intention. Int. J. Inf. Manag..

[59] Lim N. (2003). Consumers’ perceived risk: Sources versus consequences. Electron. Commer. Res. Appl..

[60] Yang Q., Pang C., Liu L., Yen D.C., Tarn J.M. (2015). Exploring consumer perceived risk and trust for online payments: An empirical study in China’s younger generation. Comput. Hum. Behav..

[61] Bashir S., Anwar S., Awan Z., Qureshi T.W., Memon A.B. (2018). A holistic understanding of the prospects of financial loss to enhance shopper’s trust to search, recommend, speak positive and frequently visit an online shop. J. Retail. Consum. Serv..

[62] Han M.C., Kim Y. (2017). Why consumers hesitate to shop online: Perceived risk and product involvement on Taobao. Com. J. Promot. Manag..

[63] Bezes C. (2016). Comparing online and in-store risks in multichannel shopping. Int. J. Retail. Distrib. Manag..

[64] Ooi K.B., Tan G.W.H. (2016). Mobile technology acceptance model: An investigation using mobile users to explore smartphone credit card. Expert Syst. Appl..

[65] Wang E.S.T., Chou N.P.Y. (2014). Consumer characteristics, social influence, and system factors on online group-buying repurchasing intention. J. Electron. Commer. Res..

[66] Kim J., Ma Y.J., Park J. (2009). Are US consumers ready to adopt mobile technology for fashion goods? An integrated theoretical approach. J. Fash. Mark. Manag..

[67] Bollen K.A., Pearl J. (2013). Eight myths about causality and structural equation models. Handbook of Causal Analysis for Social Research.

[68] Pavlou P.A., Fygenson M. (2006). Understanding and predicting electronic commerce adoption: An extension of the theory of planned behavior. MIS Q..

[69] Hair J.F., Ringle C.M., Sarstedt M. (2011). PLS-SEM: Indeed a silver bullet. J. Mark. Theory Pract..

[70] Podsakoff P.M., Organ D.W. (1986). Self-reports in organizational research: Problems and prospects. J. Manag..

[71] Allison P.D. (1998). Multiple Regression: A Primer.

[72] Wang J., Wang X. (2019). Structural Equation Modeling: Applications Using Mplus.

[73] Bagozzi R.P., Yi Y. (1988). On the evaluation of structural equation models. J. Acad. Mark. Sci..

[74] Chin W.W. (1998). Commentary: Issues and opinion on structural equation modeling. MIS Q..

[75] Fornell C., Larcker D.F. (1981). Structural equation models with unobservable variables and measurement error: Algebra and statistics. J. Mark. Res..

[76] Shaikh A.A., Karjaluoto H. (2015). Mobile banking adoption: A literature review. Telemat. Inform..

[77] Sun X., Yan W., Zhou H., Wang Z., Zhang X., Huang S., Li L. (2020). Internet use and need for digital health technology among the elderly: A cross-sectional survey in China. BMC Public Health.

[78] Grimes G.A., Hough M.G., Mazur E., Signorella M.L. (2010). Older adults’ knowledge of internet hazards. Educational Gerontology.

[79] Liao C., Liu C.-C., Chen K. (2011). Examining the impact of privacy, trust and risk perceptions beyond monetary transactions: An integrated model. Electron. Commer. Res. Appl..

[80] Kuo R.Z. (2020). Why do people switch mobile payment service platforms? An empirical study in Taiwan. Technol. Soc..

[81] Fung H.H., Carstensen L.L., Lutz A.M. (1999). Influence of time on social preferences: Implications for life-span development. Psychol. Aging.

[82] Jiang Y., Wang X., Yuen K.F. (2021). Augmented reality shopping application usage: The influence of attitude, value, and characteristics of innovation. J. Retail. Consum. Serv..

[83] Wang X., Yuen K.F., Wong Y.D., Teo C.C. (2018). An innovation diffusion perspective of e-consumers’ initial adoption of self-collection service via automated parcel station. Int. J. Logist. Manag..

[84] Cimperman M., Brenčič M.M., Trkman P. (2016). Analyzing older users’ home telehealth services acceptance behavior—Applying an Extended UTAUT model. Int. J. Med. Inform..

[85] Trafimow D., Finlay K.A. (1996). The importance of subjective norms for a minority of people: Between subjects and within-subjects analyses. Personal. Soc. Psychol. Bull..

[86] Yazdanpanah M., Forouzani M. (2015). Application of the Theory of Planned Behaviour to predict Iranian students’ intention to purchase organic food. J. Clean. Prod..

[87] Filieri R., Lin Z. (2017). The role of aesthetic, cultural, utilitarian and branding factors in young Chinese consumers’ repurchase intention of smartphone brands. Comput. Hum. Behav..

[88] Chen K., Chan A.H. (2014). Predictors of gerontechnology acceptance by older Hong Kong Chinese. Technovation.

[89] Gebhardt E., Thomson S., Ainley J., Hillman K. (2019). Introduction to gender differences in computer and information literacy. Gend. Differ. Comput. Inf. Lit..

